# The Potential Role of MicroRNA‐124‐3p in Growth, Development, and Reproduction of *Schistosoma japonicum*

**DOI:** 10.3389/fcimb.2022.862496

**Published:** 2022-04-13

**Authors:** Xue Zhou, Yang Hong, Zheng Shang, Asmaa M. I. Abuzeid, Jiaojiao Lin, Guoqing Li

**Affiliations:** ^1^ Guangdong Provincial Key Laboratory of Zoonosis Prevention and Control, College of Veterinary Medicine, South China Agricultural University, Guangzhou, China; ^2^ National Reference Laboratory for Animal Schistosomiasis, Shanghai Veterinary Research Institute, Chinese Academy of Agricultural Sciences, Shanghai, China; ^3^ Parasitology Department, Faculty of Veterinary Medicine, Suez Canal University, Ismailia, Egypt

**Keywords:** *Schistosoma japonicum*, sja-miR-124-3p, qRT-PCR, reproduction, development, *sjDDX1* silencing

## Abstract

The microRNA‐124‐3p plays an important role in regulating development and neurogenesis. Previous microRNA sequencing analyses of *Schistosoma japonicum* revealed sja-miR-124-3p differential expression patterns in schistosomes from different hosts and at different developmental stages. This study explores the regulatory role of sja-miR-124-3p in *S. japonicum* development and reproduction. Quantitative reverse-transcription PCR (qRT-PCR) showed that the expression level of sja-miR-124-3p in *S. japonicum* from resistant hosts, such as *Microtus fortis*, and unsuitable hosts, such as rats and water buffalo, was significantly higher than that in mice and yellow cattle at the same developmental stage. Overexpressing sja-miR-124-3p in infected mice led to a hepatic egg reduction rate of 36.97%, smaller egg granulomas in the livers, increased liver weight, subsided hepatocyte necrosis, and diminished inflammatory cell infiltration. The width of female worms increased but decreased in males. The vitelline cells were irregular, swollen, or fused. The teguments and ventral sucker of males and females were swollen and broken, but the morphological changes were particularly notable in males. qRT-PCR and dual-luciferase reporter assay system were used to confirm the *in-silico*-predicted target genes, *S. japonicum* DEAD-box ATP-dependent RNA helicase 1 (*sjDDX1*) and DNA polymerase II subunit 2 (*sjPOLE2*). Our results showed that RNA interference (RNAi)-mediated *sjDDX1* silencing in mice provided a 24.55% worm reduction rate and an 18.36% egg reduction rate, but the difference was not significant (*p* > 0.05). Thus, our findings suggest that sja-miR-124-3p has an important role in growth, development, and reproduction in *S. japonicum*. All these results will greatly contribute toward providing important clues for searching vaccine candidates and new drug targets against schistosomiasis.

## 1 Introduction

Schistosomiasis is a major parasitic zoonosis in tropical and subtropical countries, characterized by high infection and mortality rates (LoVerde, 2019). *Schistosoma japonicum* is a dioecious trematode and a mature female worm lays over thousands of eggs per day. Eggs deposited in the host liver and intestinal wall result in the formation of granulomas and liver fibrosis and cumulatively lead to serious pathological damage (Cheever et al., 1994; King and Dangerfield-Cha, 2008; Vale et al., 2017). By 2019, at least 236.6 million people in the world required preventive treatment for schistosomiasis (WHO, 2021). Praziquantel (PZQ) is still the drug of choice for treating *Schistosoma* infections. Although PZQ is an effective therapeutic drug for adults of *S. japonicum*, the lethal effect on schistosomula is far less than that on adults. The wide application of praziquantel has led to the gradual emergence of drug resistance (Vale et al., 2017). There is still no safe and efficient schistosomiasis vaccine available in the field.

The susceptibility of various mammals to *S. japonicum* is different, mainly in the infection degree and adult worm development and egg-laying rate. For example, only skin, lung, and hepatic stage schistosomula are formed in *Microtus fortis* (a natural non-permissive host), and most schistosomula die within 2 weeks (He et al., 2001). On the one hand, the adult worm development rate in non-suitable hosts, such as rats and equines, ranges between 5% and 20%, and the phenomenon of self-cure occurs around 1 year after *S. japonicum* infection in buffaloes and pigs (Johansen et al., 2000; Li et al., 2014). On the other hand, suitable hosts, such as yellow cattle, mice, sheep, and rabbits, generally show an adult worm development rate between 40% and 70% (Li et al., 2015). *Schistosoma japonicum* has a complex life cycle, and the growth of schistosomes in different development stages and different host environments is accurately regulated by gene expression. Clarifying the growth and development mechanism in *S. japonicum* can provide new ideas for screening schistosomiasis vaccine candidates or new drug targets.

MicroRNAs (miRNAs) are endogenous, non-coding single-stranded small RNAs (18–25 nucleotides), which regulate many biological processes, including embryonic development, cell division, cell differentiation, cell apoptosis, immune metabolism, and disease occurrence (Lee et al., 1993; Shcherbata et al., 2006; Han et al., 2013; Jeker and Bluestone, 2013; Gong et al., 2016; Zhang et al., 2021). MicroRNAs are considered potential targets for diagnosing and treating parasitic diseases due to their specific and complex regulatory effects (Mu et al., 2020). *Schistosoma japonicum* miRNA presented differential expression patterns in worms at different developmental stages, sexes, and hosts (Xue et al., 2008; Huang et al., 2009; Hao et al., 2010; Cai et al., 2011; Han et al., 2015a; Han et al., 2015b; Zhu et al., 2016; Yu X., et al., 2019).

miR-124-3p and miR-124-5p are mature miRNAs processed from the 3′ and 5′ ends of miR-124 pre-miRNA. miR-124 plays a key regulatory role in cell differentiation and the nervous system and inhibits cell proliferation in medulloblastoma (Makeyev et al., 2007; Li et al., 2009). Additionally, miR-124 may regulate ovarian and sperm development, promoting ovarian development by suppressing *Sox9* (SRY-Box Transcription Factor 9) gene expression in gonads of mice during sex determination (Real et al., 2013). miR-124 also participates in caveolin-independent vesicle trafficking and regulates the expression of sperm acrosome-related protein (flotillin-2) in mice (Wu et al., 2015). These studies suggest that miR-124 may regulate the growth, development, gender differentiation, and reproduction of schistosomes (Hao et al., 2010; Marco et al., 2013). Previous sequencing studies showed that the expression level of sja-miR-124-3p varied among worms from different developmental stages. sja-miR-124-3p expression was significantly lower in schistosomula than in cercariae (Xue et al., 2008; Hao et al., 2010; Cai et al., 2011). However, significantly higher sja-miR-124 expressions were detected in immature worms compared with adults (Huang et al., 2009), in males compared with females (Cai et al., 2011; Zhu et al., 2016), and in single-sex infecting stunted females compared with bisexual infecting mature females (Han et al., 2020). Moreover, the expression level of sja-miR-124-3p in adult schistosomes from the less suitable host (water buffalo) was always higher than that from the suitable host, yellow cattle (Yu X., et al., 2019). The expression of sja-miR-124-3p in 10-day-old schistosomula from less suitable host Wistar rats was over 10 times than that from BALB/c mice (Han et al., 2015a). Thus, we speculate that sja-miR-124-3p and some of its target genes may play an important role in the growth, development, reproduction, and survival of *S. japonicum*. However, little is known about the underlying mechanisms.

In this study, we predicted and identified sja-miR-124-3p target genes. The expression differences of sja-miR-124-3p in worms from different developmental stages retrieved from hosts with different susceptibilities to infection by *S. japonicum* were analyzed and compared by quantitative reverse-transcription PCR (qRT-PCR). The effects of sja-miR-124-3p and its target genes on worm growth, development, and oviposition were studied *in vivo* and *in vitro via* silencing or overexpressing the tested miRNA or gene. Our results provide the basis for further analyzing the biological function of sja-miR-124-3p and target genes in *S. japonicum* growth and development and also provide a novel idea for identifying vaccine candidates or new drug targets for schistosomiasis.

## 2 Materials and Methods

### 2.1 Animal Challenge and Worm Collection

Six-week-old specific pathogen-free (SPF) male BALB/c mice, Kunming mice, Sprague–Dawley (SD) rats, and reed voles (*M. fortis*) (70–80 g) were used. BALB/c mice, Kunming mice, and SD rats were purchased from the Shanghai JSJ Experimental Animal Co., Ltd. (Shanghai), while *M. fortis* was purchased from the Shanghai Laboratory Animal Center, the Chinese Academy of Sciences (CAS). *Schistosoma japonicum* (Chinese Mainland strain, Anhui isolate) cercariae and *S. japonicum* adult worms from yellow cattle and water buffalo were provided by the National Reference Laboratory of Animal Schistosomiasis, Shanghai Veterinary Research Institute, the Chinese Academy of Agricultural Sciences (CAAS). All animals were housed in a pathogen-free facility with controlled temperature and humidity, provided with water and food *ad libitum*, and maintained following the Regulations for the Administration of Affairs Concerning Experimental Animals, China. All animal experiments were approved by the Animal Care and Use Committee of Shanghai Veterinary Research Institute, CAAS (Permit ID number: SHVRI-SZ-20190620-02).

### 2.2 Expression Analysis of sja-miR-124-3p and the Predicted Target Genes by qRT−PCR

For analyzing the expression of sja-miR-124-3p and its target genes in different developmental stages of *S. japonicum*, 24 male Kunming mice and SD rats were randomly divided into four groups with six animals each. These animals were infected with *S. japonicum* cercariae by the abdominal patch method, according to **Supplementary Table 1**. The animals were sacrificed at 10, 20, 30, or 40 days (d) post-infection (p.i.), and schistosomula and adult worms were collected from the hepatic portal vein. For analyzing the expression of sja-miR-124-3p in worms of *S. japonicum* from different susceptible hosts, 10 *M. fortis* and Kunming mice were percutaneously infected with 2,000 and 200 *S. japonicum* cercariae, respectively. These animals were sacrificed at 10 d p.i., and schistosomula were isolated from the hepatic portal vein. All collected worms were washed three times with phosphate-buffered saline (PBS). Then, equal amounts of parasites were mixed with RNAlater (Ambion, Carlsbad, CA, USA) in several sterile cryopreservation tubes and stored at −80°C until further use. The 56-day-old adult worms collected from yellow cattle and water buffalo were stored at −80°C in RNAlater and provided by the National Reference Laboratory for Animal Schistosomiasis, Shanghai Veterinary Research Institute, CAAS (Yu X. et al., 2019).

Schistosome miRNAs were extracted using the miRcute miRNA Isolation Kit (Tiangen, Beijing, China). Then, the first-strand cDNA was reverse-transcribed using the miRcute enhanced miRNA cDNA kit (Tiangen, Beijing, China). The qRT-PCR of miRNAs was performed using the miRcute Plus miRNA Detection Kit (SYBR Green, Tiangen, Beijing, China) and Roche LightCycler 480 real-time PCR system. The expression levels of sja-miR-124-3p were detected by qRT-PCR using sja-U6 as an internal reference control. Each experiment was performed in duplicate in 96-well plates and repeated three times. Forward primers of sja-miR-124-3p and sja-u6 were 5′-CGCTAAGGCACGCGGTGAATGTCA-3′ and 5′-CGGCGGTACATACTAAAAT-3′, respectively. Universal reverse primer provided by the miRcute Plus miRNA Detection Kit was used in all reactions. The reaction mixture (20 μl total volume) comprised of 2× miRcute Plus miRNA PreMix (with SYBR and ROX) (10 μl), forward and reverse primer (10 µM, 0.4 μl of each), miRNA cDNA (1 μl), and RNase-free ddH_2_O (8.2 μl). The cycling conditions were as follows: 95°C for 15 min, 94°C for 20 s, and 60°C for 34 s, for 45 cycles. All samples were assessed in triplicate. Relative expression in each sample was evaluated by the comparative threshold cycle (2^−ΔΔct^) method.

Total RNA of *S. japonicum* was extracted using RNAiso Plus reagent (TaKaRa, Dalian, China) following the manufacturer’s instructions. Isolated RNA was quantified using the NanoDrop 2000 spectrophotometer (Thermo Fisher Scientific, Waltham, MA, USA). Total RNA was then reverse-transcribed into cDNA using the SuperScript III Revtranscript (Invitrogen, Carlsbad, CA, USA). Expression levels of target genes of sja-miR-124-3p were validated by qRT-PCR using the Roche LightCycler 480 real-time PCR system and ChamQ SYBR^®^ qPCR Master Mix (Vazyme Biotech Co., Nanjing, China) following the manufacturer’s instructions. Primers for target gene *sjDDX1* were as follows: 5′-GGTGGTGGACCTAATTGTCGT-3′ (forward) and 5′-GCTTTACGTTCCGGTGGACT-3′ (reverse). Primers for target gene *sjPOLE2* were 5′-TATGAGGGACTGCTTCCCGA-3′ (forward) and 5′-GACGTGAAACCTCGGGTGAT-3′ (reverse). sja-β-Actin was selected as a housekeeping control for normalization using the following primers: 5′-CGCTGTATTCCCCTCCATCG-3′ (forward) and 5′-CCAGTTGGTAACAATGCCATGT-3′ (reverse). Twenty-microliter qRT-PCR reactions contained 2× ChamQ SYBR qPCR Master Mix (10 μl), forward and reverse primers (10 µM, 0.4 µl of each), *S. japonicum* cDNA (1 µl), and ddH_2_O (8.2 µl). qRT-PCR cycling programs were predenaturation at 95°C for 30 s, 40 cycles of 95°C for 10 s and 60°C for 30 s, and melting curve stage at 95°C for 15 s, 60°C for 60 s, and 95°C for 15 s. All reactions were done in triplicate. Relative expression was analyzed by the comparative threshold cycle (2^−ΔΔct^) method (Livak and Schmittgen, 2001). The Student’s *t*-test (Statistica 10) was used to compare gene expression data between groups. Significant differences were considered when *p*-value (*p*) <0.05 and fold change ≥2.

### 2.3 Transfection of miRNAs *In Vitro*

Adult schistosomes collected from BALB/c mice, infected with *S. japonicum* cercariae 42 days post-infection, were cultured in complete DMEM/F12 medium (Dulbecco’s modified Eagle medium/nutrient mixture F-12) supplemented with 10% fetal bovine serum (Gibco, USA) at 5% CO_2_, 37°C. The worms were divided into four groups with six pair worms each. The cultured schistosomes were separately electroporated with 1 OD (optical density value) of sja-miR-124-3p mimic (GenePharma Inc., Shanghai, China), sja-miR-124-3p inhibitor (GenePharma), scramble NC (negative control), or PBS at 125 V for 24 μs. After 72 h of treatment, worms were collected, and total RNA extraction and reverse transcription were performed as in Section 2.2.

### 2.4 Interference of the Expression of sja-miR-124-3p in BALB/c Mice

**Figure 1:**
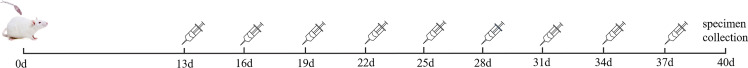
Schedule of the test for the regulation role of sja-miR-124-3p in mice.

BALB/c mice (*n* = 20) were percutaneously infected with 40 *S. japonicum* cercariae and randomly divided into four groups. On the 13th day after *S. japonicum* infection, the four groups were injected with 2 OD of sja-miR-124-3p agomir (mimic), sja-miR-124-3p antagomir (inhibitor), scramble NC in 200 μl PBS, or 200 μl PBS only *via* tail vein, once every 3 days for 9 consecutive times (see **Figure 1**). The animals were sacrificed at 9 d p.i., and adult worms were perfused from the hepatic portal vein. The worm development rate was calculated according to Formula 1. The worms were cultured in complete DMEM/F12 medium supplemented with 10% fetal bovine serum (Gibco, USA) at 4°C to separate male and female worms. Eight male and female worms from each group were taken for total RNA isolation and qPCR analysis as in Section 2. The remaining worms were used for morphological observations.


(Formula 1) $$Wormdevelopmentrate(\%)=100\times \left(\frac{thenumberofwormrecovery}{thenumberofinfectedwithcercariae}\right)$$

### 2.5 Histopathological Examination

One cubic centimeter of the fresh liver right lobe of each mouse was harvested and fixed in 4% paraformaldehyde for 2 h at room temperature and 48 h at 4°C, washed with ddH_2_O, dehydrated in gradient ethanol concentrations (30%–100%), cleared with xylene for 2 h, and embedded in paraffin wax. Following slicing, tissue sections (3 μm) were dewaxed using xylene and rinsed in 100%–30% ethanol and then in ddH_2_O. Sections were stained with hematoxylin and eosin (HE) stain, dehydrated in 95% and 100% ethanol, cleared with xylene, and mounted onto glass slides with neutral gum for microscopic observation.

### 2.6 Liver Egg Counting and Miracidia Hatching

The liver of each mouse was weighed and homogenized in 15 ml cold 2% NaCl solution in a tissue homogenizer, and then the volume was fixed to 20 ml. Eggs in 1 ml homogenate were counted after digestion with 5% NaOH in a water bath (56°C) for 10 min. The egg number in a 50-μl homogenate was counted under a microscope. The average number of three smears of each sample multiplied by 800 was considered the total egg number in each liver sample. The number of eggs per gram (EPG) was calculated by dividing the total number per liver sample by liver weight. The egg reduction rate (ERR) was calculated according to Formula 2.


(Formula 2) $$ERR\left(\%\right)=100\times \left(1-\frac{arithmeticmeaneggcountsattreatmentgroup}{arithmeticmeaneggcountsatPBSgroup}\right)$$

Liver homogenate (4 ml) was mixed with dechlorinated water in a flat-bottom flask, and a thin layer of absorbent cotton was placed in the bottleneck. After incubation at 28°C for 2 h, the supernatant above the cotton layer was collected. Dechlorinated water was re-added to the flask, and the supernatant was collected again after 2 h. The collected supernatants were mixed with two iodophor drops to fix and stain the miracidia. After centrifugation at 3,000*g* for 5 min, the solution was resuspended to 2 ml. A 50-μl resuspension was taken, and the number of miracidia was counted under the microscope. Each sample was counted three times, and the average of three counts was multiplied by 80. The miracidia total number in 4 ml homogenate was obtained. The total number of hatched miracidia was calculated by multiplying the results in the previous step by 5. Calculation methods of hatchability of liver eggs and reduction of hatching rate of liver eggs are shown in Formulas 3 and 4.


(Formula 3) $$Hatchabilityoflivereggs\left(\%\right)=100\times \left(\frac{Thetotalnumberofmiracidia}{Thetotalnumberofeggs}\right)$$

(Formula 4) $$Reductionofhatchingrateoflivereggs\left(\%\right)=100\times \left(1-\frac{arithmeticmeanhatchabilityoflivereggsattreatmentgroup}{arithmeticmeanhatchabilityoflivereggsatPBSgroup}\right)$$

### 2.7 Measurement of the Worm Size and Observation on Reproduction System of *Schistosoma japonicum*

The male and female worms were washed three times with PBS, then fixed in AFA fixing solution at room temperature for 13 h. After gradient dehydration in 30%, 50%, 70%, 95%, and 100% ethanol, worms were stained with 2% carmine hydrochloride solution, decolorized with 1% hydrochloric acid-alcohol solution, and cleared with xylene. The length and width of individual *S. japonicum* female and male adult worms (40-day-old) were measured by a Leica fluorescent inverted microscope. The worms’ reproductive systems were observed with a Nikon C1Si laser scanning confocal microscope.

### 2.8 Ultrastructural Analysis by Scanning Electron Microscopy and Transmission Electron Microscopy

For scanning electron microscopy (SEM), worms were fixed with 2.5% glutaraldehyde in PBS (pH 7.4) for 24 h at room temperature. After rinsing three times in PBS, the worms were stored in the same buffer at 4°C until use. Before SEM examination, the flukes were washed twice with double-distilled water, dehydrated in ascending ethanol concentrations, rinsed in isoamyl acetate twice, and air-dried. Worms were placed on aluminum stubs, sputter-coated with 20 nm gold particles, and observed in a high-resolution SEM (Philips XL30 ESEM) at an accelerating voltage of 5 kV.

For transmission electron microscopy (TEM), worms were fixed with 2.5% glutaraldehyde/PBS (pH 7.4) at 4°C, washed with PBS, fixed with 1% osmic acid, washed with distilled water, dehydrated with gradient concentrations of ethanol, and embedded in epoxy resin. Next, 50-nm-thick sections were processed and labeled with uranyl acetate and lead citrate and then observed under TEM (Tecnai G2 Spirit BioTwin; FEI, Hillsboro, OR, USA).

### 2.9 Prediction of Target Genes

TargetScan (Agarwal et al., 2015), RNAhybrid (Rehmsmeier et al., 2004), miRDB (Chen and Wang, 2020), miRTarBase (Huang et al., 2020), and miRmap (Vejnar and Zdobnov, 2012) were used for predicting sja-miR-124-3p target genes in mRNAs [5′-untranslated region (UTR), coding sequence (CDS), or 3′-UTR] (Cai et al., 2016). Gene sequences were downloaded from the NCBI website (bioproject ID: PRJNA520774) and WormBase ParaSite v.10. The target genes of sja-miRNA-124-3p (score ≥ 150 and energy ≤ 20) were firstly predicted by the miRanda website, and the results showed that sja-miRNA-124-3p had 70 target genes. Then, 70 predicted target genes were compared and screened. NCBI and UniProt sequences were used to exclude those target genes whose gene information was completely unknown, and 42 target genes that had been studied in other species were screened. We chose genes related to development and reproduction, whose similarity was reported in our previous studies. We transfected the cultured adults with sja-miR-124-3p mimics, detected the expression levels of some target genes related to reproductive development function by qRT-PCR, and screened out genes negatively correlated with the expression levels of sja-miR-124-3p. Subsequently, the RNAhybrid website was mainly used to predict the target sites of miRNA in the exon region of the above target genes, and the binding site with the lowest MFE value was selected in the final displayed results. Finally, the double luciferase reporter gene assay was used to determine the targeting relationship between sja-miR-124-3p and target genes (*sjDDX1* and *sjPOLE2*).

### 2.10 Dual−Luciferase Reporter Assay

The sequence of 200 bp before and after the 3′-UTR binding site of the target gene and the corresponding sja-miR-124-3p mutants (GenePharma Inc., Shanghai, China) were cloned and inserted into the 3′-UTR of pmirGLO luciferase vector (Promega, Madison, WI, USA). In 24-well plates, 293 T cells were cultured to approximately 70% confluence and then co-transfected with either wild-type or mutant luciferase reporter vector (2 μg) and either sja-miR-124-3p mimic miRNAs or negative control (NC) (2 μg) with Lipofectamine 2000 reagent. Luciferase activity was measured using the Dual-Luciferase Reporter Assay System (Promega, Madison, WI, USA) after transfection for 48 h. Both Renilla and firefly luciferase activities were detected on a multifunctional enzyme labeling instrument using the Dual-Luciferase Reporter Assay System. The relative luciferase activity was normalized to the Renilla luciferase activity. Each experiment was biologically repeated three times.

### 2.11 Target Gene Silencing Through RNA Interference *In Vitro* and *In Vivo*

Five target genes of *sjDDX1*-specific small interfering RNAs (siRNAs) (si5, si6, si7, si8, and si9) and a non-specific siRNA were chemically synthesized by GenePharma Inc. The siRNA sequences are as follows: sjDDX1-si-5: sense (5′–3′) GGUCGUGGUUGUUACAAUAdTdT, antisense sense (5′–3′) UAUUGUAACAACCACGACCdTdT; sjDDX1-si-6: sense (5′–3′) GGAGAUAUGGUUUCUAGAAdTdT, antisense (5′–3′) UUCUAGAAACCAUAUCUCCdTdT; sjDDX1-si-7: sense (5′–3′) GAUGUUAAGAGAUUAGCUAdTdT, antisense (5′–3′) UAGCUAAUCUCUUAACAUCdTdT; sjDDX1-si-8: sense (5′–3′) GCUUAUGUCCAGAAACAAAdTdT, antisense (5′–3′) UUUGUUUCUGGACAUAAGCdTdT; sjDDX1-si-9: sense (5′–3′) GAUAGAUUUGAAAGGUCAAdTdT, antisense (5′–3′) UUGACCUUUCAAAUCUAUCdTdT; SjPOLE2-158: sense (5′–3′) CCGGAUGCUUCUCAGUAUATT, antisense (5′–3′) UAUACUGAGAAGCAUCCGGTT; SjPOLE2-429: sense (5′–3′) CGUCUGAGAAUCGCUUAAUTT, antisense (5′–3′) AUUAAGCGAUUCUCAGACGTT; SjPOLE2-662: sense (5′–3′) GCAGCACCUUUAGAUACAATT, antisense (5′–3′) UUGUAUCUAAAGGUGCUGCTT; and NC: sense (5′–3′) UUCUCCGAACGUGUCACGUTT, antisense (5′–3′) ACGUGACACGUUCGGAGAATT. Six pairs of adult schistosomes in each group were separately electroporated with 1 OD of *sjDDX1* siRNA, scramble NC, or PBS only at 125 V for 24 μs. The worms were collected 3 days post-treatment for qRT-PCR analysis. Egg counts were carried out every 24 h post-treatment, and the egg reduction rate was calculated. Approaches of total RNA isolation, reverse transcription, and qRT-PCR were the same as in Sections 2.2 and 2.3.

BALB/c mice (*n* = 15) were percutaneously infected with 80 *S. japonicum* cercariae and randomly divided into three groups. Beginning from the 14th day after *S. japonicum* infection, 2 OD *sjDDX1* siRNA, scramble NC in 200 μl PBS, or 200 μl PBS only was injected into the tail vein of each BALB/c mice, once every 7 days for four consecutive times. The animals were sacrificed at 42 d p.i., adult worms were perfused from the hepatic portal vein, and the worm number in each group was counted.

### 2.12 Statistical Analysis

Data were analyzed using IBM SPSS software package version 23.0 (IBM Corp, Armonk, NY, USA). Comparisons between the studied groups were analyzed using *F*-test (ANOVA) and *post-hoc* test (Tukey) for pairwise comparisons. The significance of the obtained results was judged at the 5% level.

## 3 Results

### 3.1 Comparison of the Expression of sja-miR-124-3p Between Schistosomes at Different Developmental Stages From Mice and Rats

**Figure 2:**
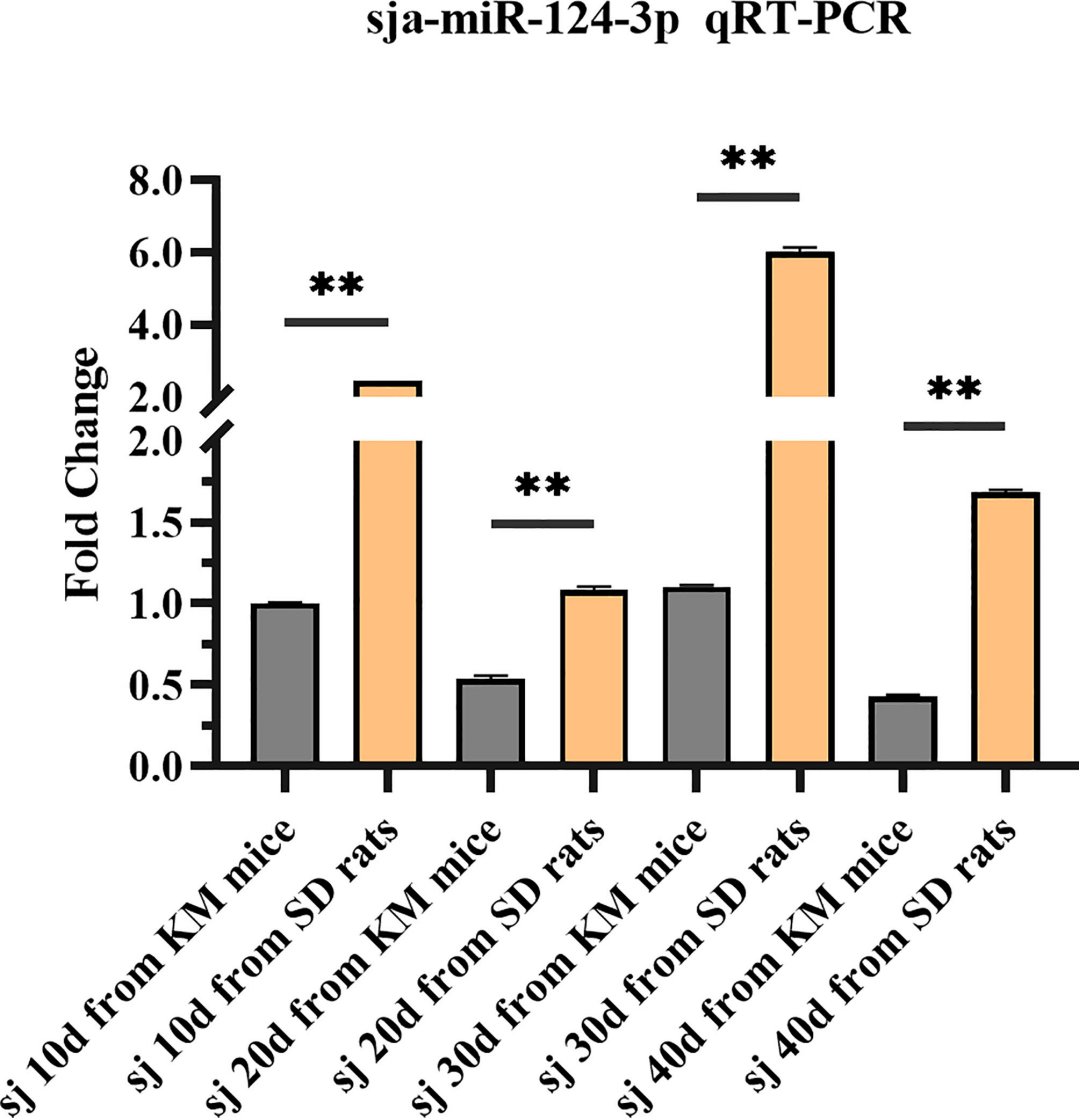
Analysis of the expression level of sja-miR-124-3p in different developmental stages of schistosomes in mice and rats by qPCR. Statistically significant differences between mice and rats are shown by ** (*p* < 0.01).

sja-miR-124-3p was expressed in all four stages (10, 20, 30, 40 days) of *S. japonicum*. The expression level in rat schistosomes was significantly higher (*p* < 0.01) than in mice schistosomes at the same growth stage. The former was 2.457, 2.022, 5.452, and 3.945 times higher than the latter at the four stages. The sja-miR-124-3p expression level in rats was the highest in 30-day-old worms and the lowest in 20-day-old worms. The expression level of sja-miR-124-3p in 10- and 30-day-old worms from mice was higher than that in 20- and 40-day-old worms (**Figure 2**).

### 3.2 Comparison of the Expression of sja-miR-124-3p Between Schistosomes From Different Susceptible Hosts

**Figure 3:**
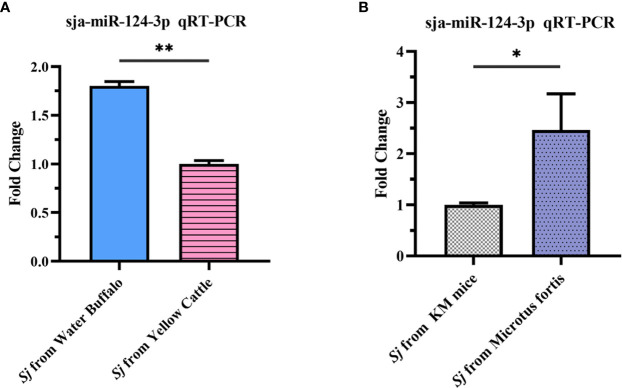
qRT-PCR analysis of the expression level of sja-miR-124-3p in *Schistosoma japonicum* collected from hosts with different susceptibilities to infection. **(A)** Adult *S. japonicum* from buffalo and cattle. **(B)** Schistosomula from KM mice and *Microtus fortis*. Statistically significant differences between the schistosome from different suitable hosts are shown by * (*p* < 0.05) and ** (*p* < 0.01).

Results showed that the expression level of sja-miR-124-3p in 56-day-old adult *S. japonicum* worms from non-susceptible host water buffalo was 1.803 times higher than that from susceptible host yellow cattle (**Figure 3A**). The expression level in 10-day-old schistosomula from the resistant host *M. fortis* was 2.43 times higher than that from the susceptible host Kunming mice (**Figure 3B**). These results showed that the expressing level of sja-miR-124-3p in worms from non-susceptible hosts is significantly higher than that from susceptible hosts (*p* < 0.05).

### 3.3 Effects of sja-miR-124-3p Overexpression and Decreased Expression on the Schistosome Development

**Figure 4:**
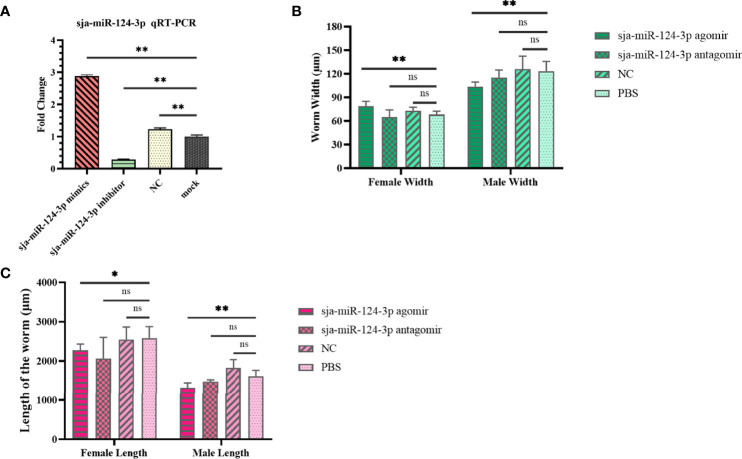
The regulating role of sja-miR-124-3p on the development of *S. japonicum in vitro* and *in vivo.* **(A)** Expression level of sja-miR-124-3p in worms transfected with mimics or inhibitor of sja-miR-124-3p *in vitro*. **(B)** Graph showing the effect on worm width. **(C)** Graph showing the effect on worm length. Statistically significant differences are shown by ns (p ≥ 0.05), * (*p* < 0.05) and ** (*p* < 0.01).

qRT-PCR results showed that when schistosomes were electroporated *in vitro* with sja-miR-124-3p mimic or inhibitor, the sja-miR-124-3p expression level was significantly higher or lower than in the NC and mock groups. These data indicate that sja-miR-124-3p mimic and inhibitor could be successfully transfected into *S. japonicum* (**Figure 4A**).

The effects of sja-miR-124-3p overexpression and decreased expression on the schistosome development in mice are shown in **Supplementary Table 2**. The worm development rate in the sja-miR-124-3p agomir, antagomir, NC, and PBS groups was 63%–67%, and no significant difference was observed. The size of the worms was measured by microscope image analysis. Worms in the sja-miR-124-3p agomir group were significantly shorter than those in the PBS group (*p* < 0.01) (**Figure 4C**). The female width in the sja-miR-124-3p agomir group was 78.886 ± 6.180 μm, wider than those in the PBS group (*p* < 0.01), while the male width was 103.832 ± 5.670 μm, narrower than those in the PBS group (*p* < 0.01). Although the injection of sja-miR-124-3p antagomir and NC also had a certain effect on the length and width of female and male worms, no significant difference was observed (*p* > 0.05) (**Figures 4B, C** **)**.

**Figure 5:**
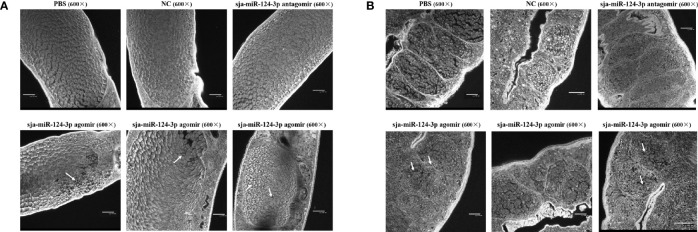
The observation of the worm reproductive system of *S. japonicum* by confocal laser scanning microscope (CLSM). **(A)** Ovary of female (×600). Some oocytes showed shrinkage or vacuolization (→). **(B)** Testis of male (600×). The edge of some testis was blurred, sperm cells were shrunken and uneven in size, and the testis cell development was out of order (→).

Confocal laser scanning microscopy showed that the male testis had clear edges and was full of sperm cells. Different developmental stages of oogonium, oocytes, and eggs were seen in the female ovary in worms from the PBS and NC groups. No obvious abnormalities in the reproductive system of male and female worms were observed in the sja-miR-124-3p antagomir group. However, in worms from the sja-miR-124-3p agomir group, the edge of some male testis was blurred, sperm cells were shrunken and uneven in size, and the testis cell development was out of order (bottom row in **Figure 5B**). Similarly, some female oocytes showed shrinkage or vacuolization and decreased number of mature oocytes and increase of immature ones, and the boundaries between oocyte stages appeared blurred in worms from the sja-miR-124-3p agomir group (bottom row in **Figure 5A**).

### 3.4 Effects of Overexpression and Decreased Expression of sja-miR-124-3p on the Mice Liver

sja-miR-124-3p agomir injection significantly increased mice liver weight (*p* < 0.01) by 27.06% compared with mice injected with PBS. The liver weight of mice treated with antagomir and scramble NC increased compared with that of the PBS injection group (*p* > 0.05) (**Supplementary Table 3**). After multiple administrations of sja-miR-124-3p agomir to infected mice, ERR was significantly decreased (*p* < 0.05) compared with PBS-injected mice. The sja-miR-124-3p antagomir and scramble NC injection groups had a certain egg reduction effect, but it was not significant (**Supplementary Table 3**).

The injection of sja-miR-124-3p agomir, antagomir, or NC could reduce the liver egg hatching rate. There was a significant difference in the liver egg hatching rate between the agomir group and the NC group (*p* < 0.05), but no significant difference was observed between the antagomir group (*p* > 0.05) and the PBS group (**Supplementary Table 3**).

**Figure 6:**
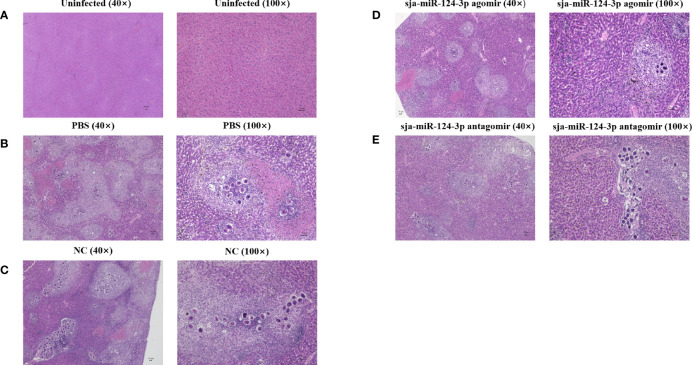
Observation of pathological liver tissue with HE staining. **(A)** The uninfected group, **(B)** the PBS group, **(C)** the NC-treated infected group, **(D)** the sja-miR-124-3p agomir-treated group, and **(E)** the sja-miR-124-3p antagomir-treated group. Bar = 50 μm.

Histopathological examination showed no hepatic pigmentation in the liver of uninfected mice. The structure of the hepatic lobule was complete and clear, and the hepatocytes were round with clear boundaries. The inflammatory cell infiltration and hepatocyte necrosis were not observed (**Figure 6A**). In *S. japonicum*-infected mice, granulomatous nodules containing single or multiple eggs and necrotic foci in the liver lobules were observed. Hepatic tissue showed coagulation clots and necrosis, with indistinct boundaries and nucleus. The central vein was surrounded by hepatocytes with vacuolar degeneration and fat vacuoles. The portal area was surrounded by lymphatic plasma cells. Inflammatory cell infiltration in the hepatic sinuses and hepatic pigmentation were detected (**Figures 6B, C, E** **)**. Compared with other groups, the liver of mice injected with sja-miR-124-3p agomir showed hepatocyte necrosis and inflammatory cell infiltration was seemingly reduced 42 days after infection (**Figure 6D**).

**Figure 7:**
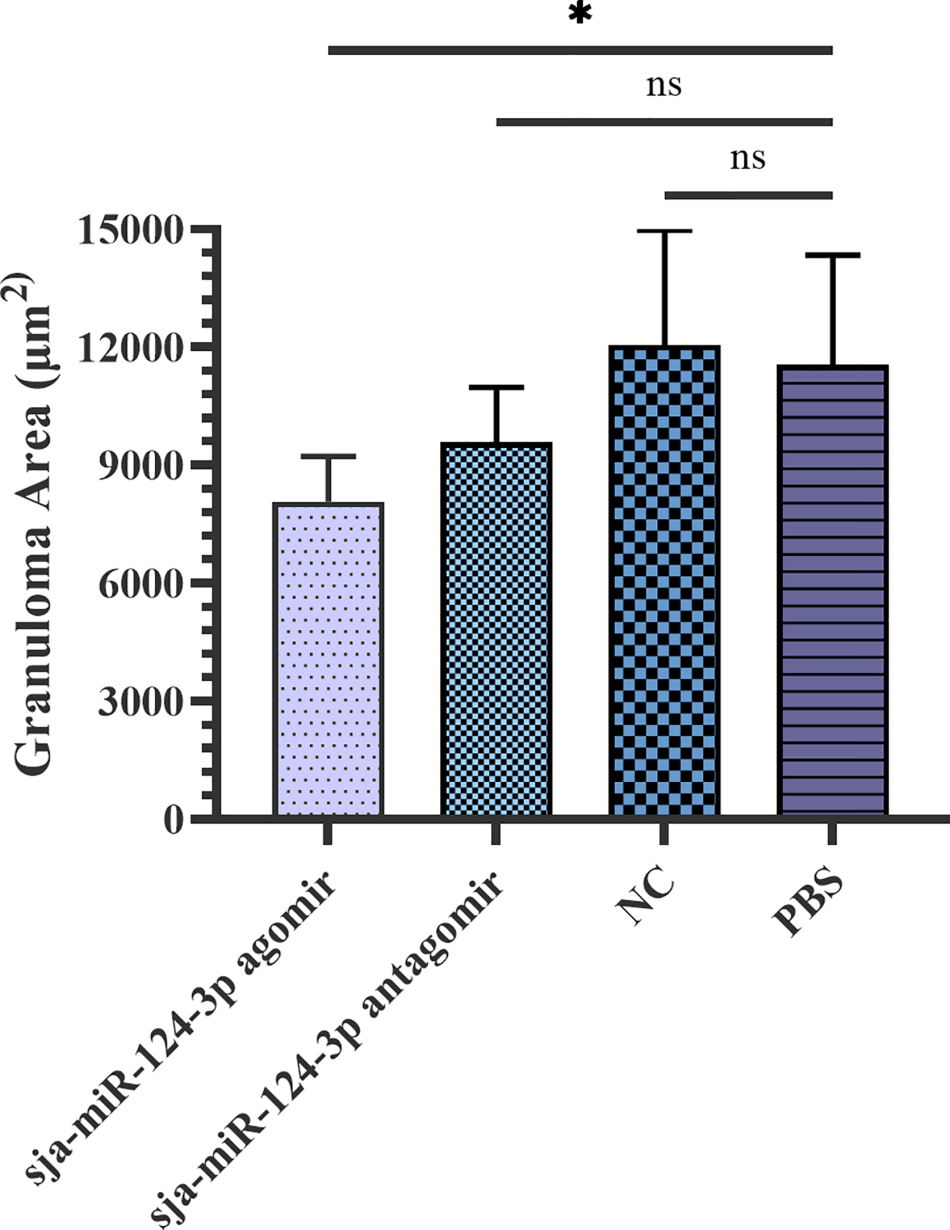
Statistical analysis of liver granuloma area of *S. japonicum*-infected mice. Statistically significant differences are shown by ns (*p* ≥ 0.05) and * (*p* < 0.05). sja-miR-124-3p antagomir: mice injected with sja-miR-124-3p inhibitor; sja-miR-124-3p: mice injected with sja-miR-124-3p; NC: mice injected with scramble NC; PBS: mice injected with PBS.

The granuloma area formed by a single *Schistosoma* egg in the liver was measured under a microscope and statistically analyzed by ImageJ. The average granuloma area was (8.08 ± 1.12) × 10^3^ μm^2^ in the sja-miR-124-3p agomir group, (9.59 ± 1.39) × 10^3^ μm^2^ in the sja-miR-124-3p antagomir group, (1.21 ± 0.29) × 10^4^ μm^2^ in the scramble NC group, and (1.16 ± 0.28) × 10^4^ μm^2^ in the PBS group. *t*-test results showed that the area of egg granulomas in the livers of the sja-miR-124-3p agomir group was significantly smaller than that of the PBS group (*p* < 0.05). However, there was no statistically significant difference in granuloma areas between the antagomir, the NC, and the PBS groups (*p* > 0.05) (**Figure 7**).

### 3.5 Ultrastructural Electron Microscopic Observation

**Figure 8:**
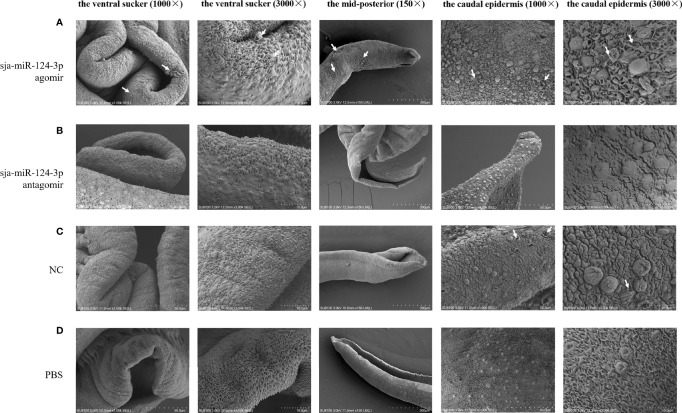
The observation of the male worms of schistosome by SEM. **(A)** sja-miR-124-3p agomir-treated group, **(B)** sja-miR-124-3p antagomir-treated group, **(C)** NC-treated group, **(D)** PBS-treated group.

SEM showed that the ventral sucker of the male worms collected from mice treated with the sja-miR-124-3p agomir was flabby and swelling. Some spines fell off and small holes, erosions, and swelling occurred on the surface of the middle and posterior part of males in varying degrees. The papillae on the tail were abnormally enlarged and increased. The boundaries of the tegumental ridges were not obvious, and fusion occurred in varying degrees. The distribution of small spines was disordered or disappeared, and the blister protrusion of males burst (**Figure 8A**). In male worms collected from mice injected with sja-miR-124-3p antagomir or PBS, the spines of the ventral sucker were arranged in order, and ridges or papillae were distributed thoroughly. The hemispherical papillae with cilium were kept plump (**Figures 8B, D** **)**. The scramble NC treatment group showed normal and plump spines of the ventral sucker, while holes, erosions, and swellings occurred on individual hemispherical papillae. The degree of ridge swelling was less apparent (**Figure 8C**).

**Figure 9:**
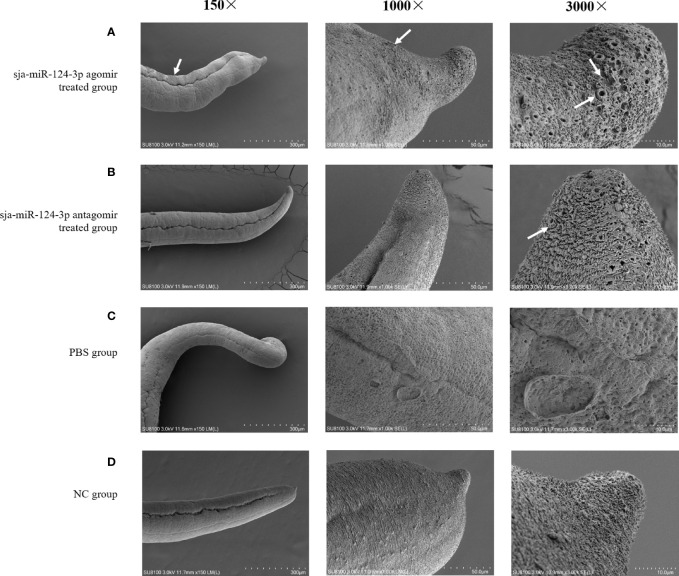
SEM observation of the mid-posterior of female worms after treatment. **(A)** sja-miR-124-3p agomir-treated group, **(B)** sja-miR-124-3p antagomir-treated group, **(C)** PBS-treated group, **(D)** NC-treated group.

In SEM analysis, *S. japonicum* adult females isolated from sja-miR-124-3p agomir-injected mice revealed variably fused tail ridges, swollen spines, and holes formed by increased tissue depression (**Figure 9**). There were increased vesicles on the tegument, broken epidermis, and syncretized pitted sheet tegument. *Schistosoma japonicum* females from the sja-miR-124-3p antagomir group also showed a small number of holes on the tail, but there were no obvious pathological changes in the spines and ridges. In female worms of the scramble NC and PBS groups, no small holes were observed on the worm surface, body spines were arranged regularly, and ridges were low, flat, and compact. Females were less seriously damaged than males, which may be due to wrapping the female in the gynecophoral canal of the male.

**Figure 10:**
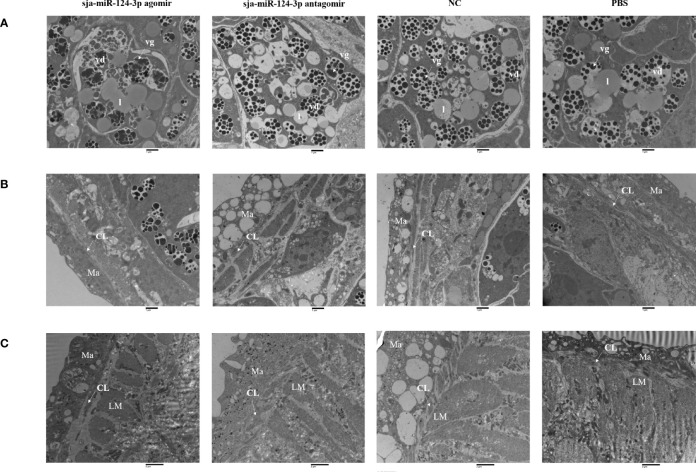
TEM observation of the integument of schistosome and yolk cells of female worms. **(A)** The yolk cells of female worms; **(B)** the integument of female worms; **(C)** the integument of male worms after miRNA product treatment. **(A, B)** Magnification of ×9,300. **(C)** Magnification of ×6,800. vd, vitelline droplet; vg, vitelline globules; l, lipid droplet; CL, circular muscle layer; LM, inner longitudinal muscle; Ma, matrix. Bar = 1 μm.

TEM of female worms from the sja-miR-124-3p agomir group showed that the size and shape of vitelline droplets (vd) in female vitelline cells were irregular and swollen. Some vitelline droplets (vd) fused, disintegrated, and dissolved. The structure was blurred, the rod secretory bodies were reduced, the tegument structure was loose, and focal matrix (Ma) dissolution and vesicle formation were detected. In the antagomir group, females had a lysed subtegumental parenchyma, lipid droplets (l) partially disappeared, and some vitelline droplets (vd) collapsed. A few vesicles on the female matrix (Ma) were observed in worms from the scramble NC or sja-miR-124-3p antagomir groups. The vitelline and lipid droplets (l) were abundant and compact in the PBS and NC groups. The tegument structure was compact, and there was no significant ultrastructural pathological change in worms from mice injected with PBS (**Figure 10**).

In male worms from the sja-miR-124-3p agomir group, the outer circular muscle partially degenerated, and the stratification was not clear. There was no significant ultrastructural pathological change in the inner longitudinal muscle (LM) and matrix (Ma). In male worms from the sja-miR-124-3p antagomir group, lysis of the inner longitudinal muscle (LM) was observed, with no significant changes in the outer circular muscle and matrix (Ma). In male worms from the NC group, the matrix (Ma) was indistinct, while the outer circular muscle and the inner longitudinal muscle (LM) had no obvious changes. The tegument structure in worms from the PBS group was compact, and no significant changes were observed (**Figure 10**).

### 3.6 Prediction and Validation of sja-miR-124-3p Target Genes

**Figure 11:**
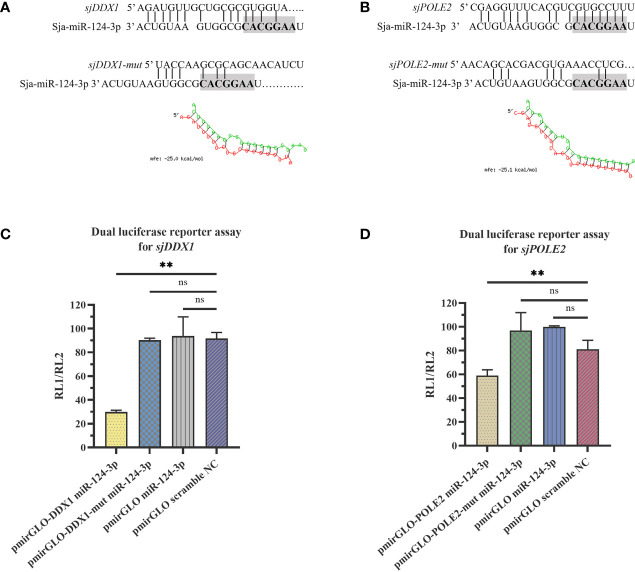
sja-miR-124-3p targeted and regulated *sjDDX1* and *sjPOLE2*. **(A, B)** The sequence of sja-miR-124-3p and the predicted binding sites with sja-miR-124-3p within the *sjDDX1* **(A)** and *sjPOLE2* **(B)** 3′ untranslated region (3′-UTR). **(C, D)** sja-miR-124-3p stimulation inhibited mRNAs in HEK 293T cells, then lysed to measure the relative luciferase activity. Statistically significant differences are shown by ns (*p* ≥ 0.05), and ** (*p* < 0.01). *n* = 3.

**Figure 12:**
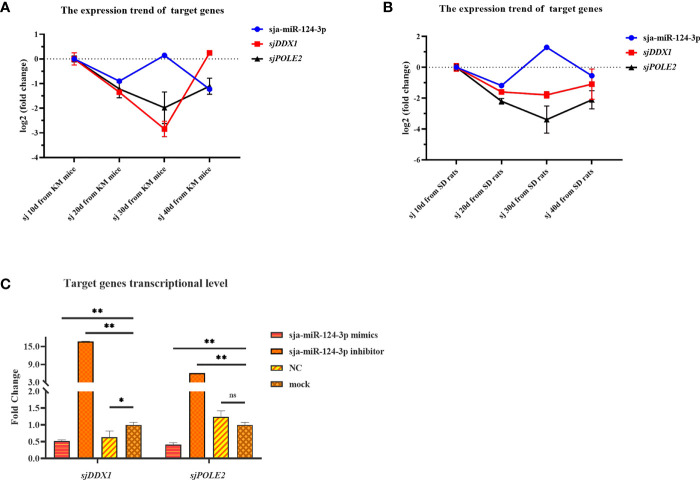
Analysis of the regulation of target genes *sjDDX1* and *sjPOLE2* by sja-miR-124-3p. **(A, B)** Gene expression trend changed in *Schistosoma japonicum* from KM mice **(A)** and SD rats **(B)**. **(C)** Compared with the PBS group, the expression levels of *sjDDX1* and *sjPOLE2* in the sja-miR-124-3p mimics group were downregulated, and the expression levels of *sjDDX1* and *sjPOLE2* in the sja-miR-124-3p inhibitor group were upregulated, while in the NC group, the levels were not significantly changed. Statistically significant differences are shown by ns (*p* ≥ 0.05), * (*p* < 0.05), and ** (*p* < 0.01).

TargetScan prediction showed that miR-124-3p could bind to the 3′-UTR region of ATP-dependent RNA helicase DDX1 (*sjDDX1*) and DNA polymerase II subunit 2 (*sjPOLE2*), with MFE values of −25.0 and −25.1 kcal/mol, respectively (**Figures 11A, B** **)**. RL1 (firefly luciferase activity)/RL2 (Renilla luciferase activity) significantly decreased when transfecting sja-miR-124-3p mimic with *sjDDX1* or *sjPOLE2* recombinant double luciferase reporter plasmid. These data indicate that sja-miR-124-3p regulated *sjDDX1* and *sjPOLE2*. Transfection of sja-miR-124-3p mimic into *S. japonicum* adult worms *in vitro* significantly lowered the expression levels of *sjDDX1* and *sjPOLE2* (*p* < 0.01) (**Figures 11C, D** **)**. In worms isolated from the host, the transcription levels of *sjDDX1* (**Figure 12A**) and *sjPOLE2* (**Figure 12B**) in worms at different developmental stages were generally opposite to that of sja-miR-124-3p. However, when the sja-miR-124-3p inhibitor was transfected, *sjDDX1* and *sjPOLE2* expression levels significantly increased (*p* < 0.01) (**Figure 12C**).

### 3.7 Effects of RNAi-Mediated *sjDDX1* Silencing on *Schistosoma japonicum*

**Figure 13:**
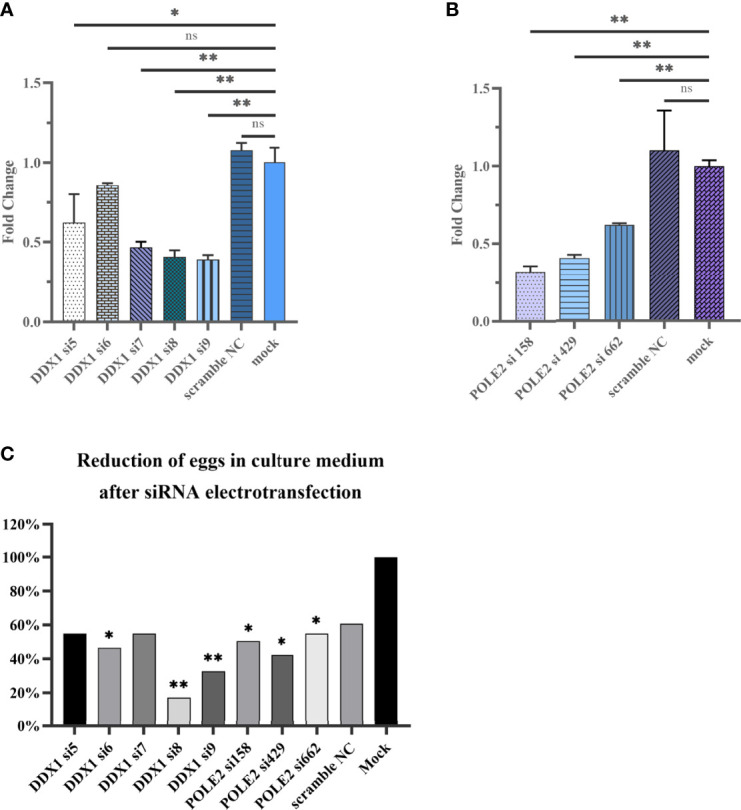
Analysis of *sjDDX1* **(A)** and *sjPOLE2* **(B)** gene expression silencing induced by RNAi. Reduction of eggs in culture medium after siRNA electrotransfection **(C)**. Each column represents the percentage of egg count compared with the mock group. Statistically significant differences are shown by ns (*p* ≥ 0.05), * (*p* < 0.05), and ** (*p* < 0.01).

Five small interfering RNAs targeting *sjDDX1* were designed and three small interfering RNAs targeting *sjPOLE2* were synthesized by Gemma gene company and electrotransfected into paired *S. japonicum* adult worms *in vitro*. Eggs were collected from the culture medium 12, 24, 48, and 72 h after electrotransfection and counted. After electrotransfected, compared with the mock group, the expression levels of *sjDDX1* in the si7, si8, and si9 groups were downregulated (*p* < 0.01) (**Figure 13A**), and the expression levels of *sjPOLE2* in the si158, si429, and si662 groups were downregulated (*p* < 0.01) (**Figure 13B**). Results showed that the egg number significantly decreased (*p* < 0.01) in the siRNA7, siRNA8, and siRNA9 treatment groups compared with untreated and irrelevant siRNA-treated worms (**Figure 13C**).

We used small interfering RNA (siRNA8) to evaluate the effect of *sjDDX1* on the development of *S. japonicum* in mice. Results showed that a 24.55% worm reduction rate and an 18.36% liver egg reduction rate were detected, but the difference was not significant compared with the PBS control group (*p* > 0.05) (**Supplementary Table 4**).

**Figure 14:**
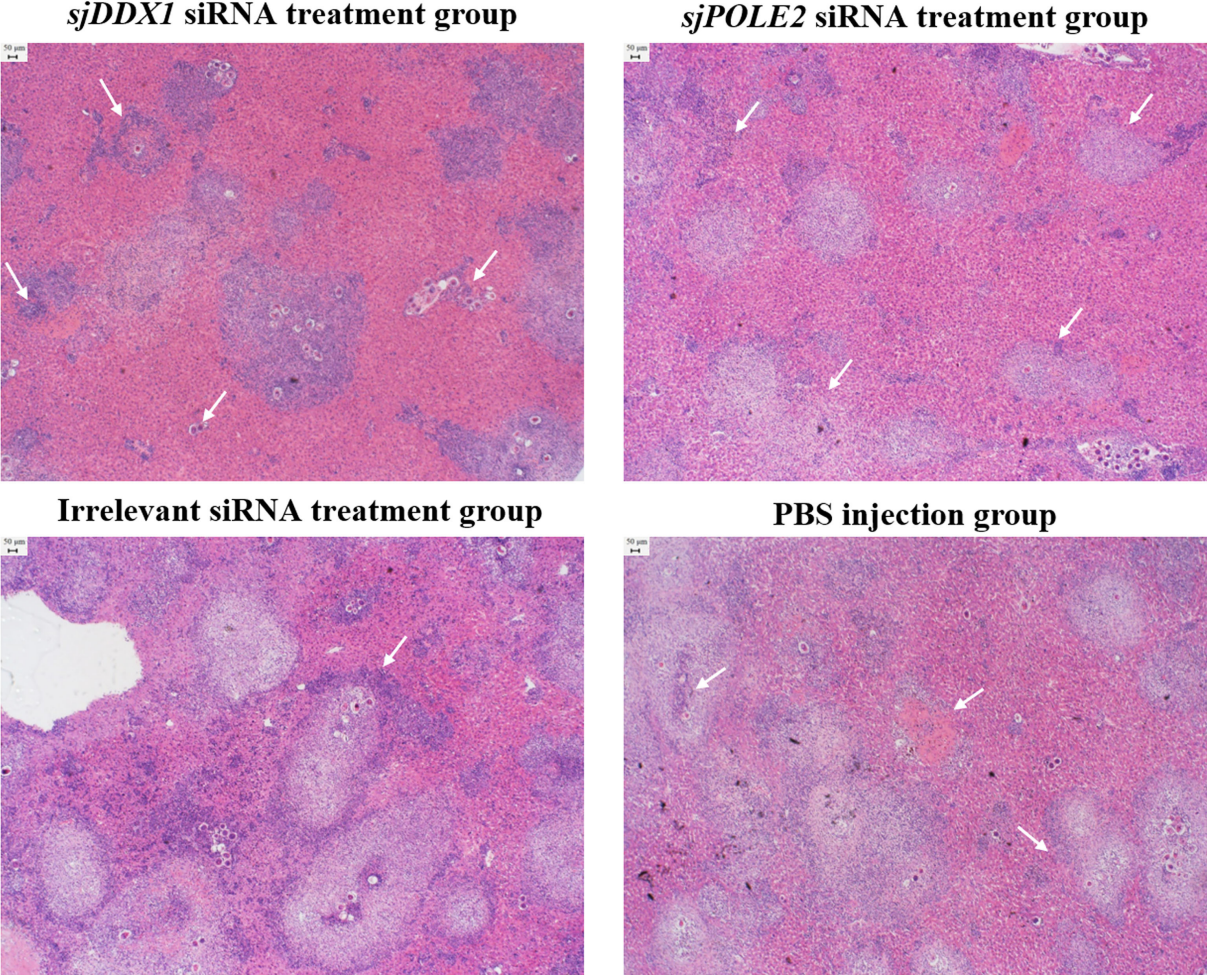
HE staining observation of the liver after *sjDDX1* and *sjPOLE2* siRNA treatment (×40). The PBS group, the irrelevant siRNA treatment group, and the *sjPOLE2* siRNA treatment group showing multiple granulomas surrounded partly or totally by degenerated ova and large cellular infiltrate. The *sjDDX1* siRNA treatment group showing the apparent reduction of hepatocyte necrosis and inflammatory cell infiltration. Bar = 50 μm.

**Figure 15:**
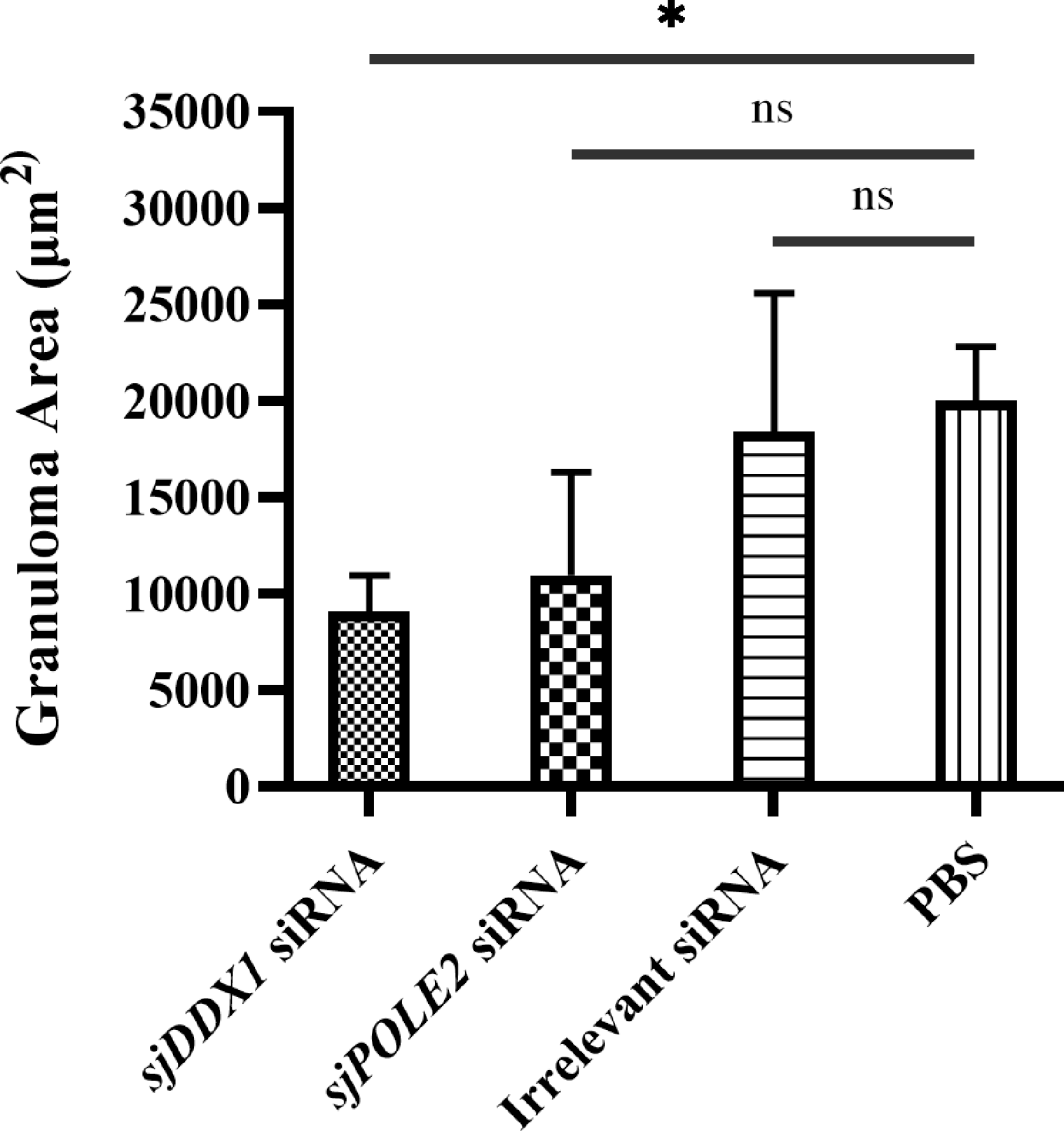
Determination of liver granuloma area of *S. japonicum*-infected mice. Statistically significant differences are shown by ns (*p* ≥ 0.05) and * (*p* < 0.05).

Hepatic pathological observations showed that the pathologic changes were milder in the *sjDDX1*-specific siRNA8 treatment group than in other schistosome infection groups (**Figure 14**). The volume of egg granuloma in liver tissue of *Schistosoma*-infected mice in the siRNA-mediated *sjDDX1* silencing group was significantly smaller than in other groups 42 days after infection. Moreover, the degree of hepatocyte necrosis and inflammatory cell infiltration was significantly reduced (**Figure 14**). We used ImageJ to measure and statistically analyze the area of granuloma around a single egg. The size of egg granulomas was significantly smaller in the *sjDDX1* siRNA injection group than in the irrelevant siRNA and PBS groups, with no significant difference between them (*p* < 0.05) (**Figure 15**).

## 4 Discussion

*Schistosoma japonicum* miRNAs are predicted to play an important role in the growth, development, and reproduction of worms (Yu J., et al., 2019). However, little is known regarding the regulation mechanism of miRNA in the development of *S. japonicum.* Understanding the biological function of miRNA in *S. japonicum* development may offer thoughts for developing a new approach to control schistosomiasis.

Previous miRNA sequencing studies showed that sja-miR-124-3p in adult schistosomes isolated from water buffalo and 10-day-old schistosomula isolated from Wistar rats was significantly higher than that from yellow cattle and BALB/c mice, respectively (Han et al., 2015a; Yu X., et al., 2019). Moreover, the sja-miR-124-3p expression level in unisexual and stunted *S. japonicum* females was significantly higher than in paired females (Han et al., 2020). In this study, we further confirmed previous data by analyzing the differential expression of sja-miR-124-3p in different developmental stages and worms isolated from different hosts. qRT-PCR results showed that the expression of sja-miR-124-3p was detected in all four stages and was significantly higher in schistosomes from rats than mice at the corresponding growth stage. In mice, the sja-miR-124-3p expression level in the 10-day-old schistosomula was higher than in the 20- and 30-day-old worms. The expression of sja-miR-124-3p was higher in 30-day-old worms than in 40-day-old worms. In schistosomes from rats, the relative expression level of sja-miR-124-3p was the highest in 30-day-old worms. We also compared sja-miR-124-3p expression in schistosomes from different hosts. Compared with mice (suitable host), 10-day-old schistosomulum from *M. fortis* (non-permissive host) had significantly overexpressed sja-miR-124-3p. sja-miR-124-3p expression level in 56-day-old adult worms from buffalo was higher than that from cattle. Compared with mice and yellow cattle, the microenvironment in rats, *M. fortis*, and water buffalo is less suitable for the growth and development of *S. japonicum*. The expression level of sja-miR-124-3p in adult or schistosomula from unsuitable hosts was higher than those from susceptible hosts. These data suggest that the growth, development, and survival of *S. japonicum* may be related to the expression level of sja-miR-124-3p.

The detected sja-miR-124-3p differential expression at different developmental stages might be explained by obvious differences in the growth rate of *S. japonicum* at different developmental stages, previously recorded by Fang et al. (2010). The slower growth rate in 1 to 8 days helps schistosomula to adapt to the host environment. The speed of growth and development increased at 8 to 12 days. The reproductive organs of worms were almost formed on the 20th day, and vigorous metabolism and rapid growth and development occur at this stage. The reproduction of females activated at the beginning of day 23, reaching the largest steady state at day 30 to 40. Metabolism was active at this stage (Huang et al., 2009). The sja-miR-124-3p expression level was high in *S. japonicum* eggs (Cai et al., 2013). Bioinformatics analysis showed that target genes of sja-miR-124-3p were mainly associated with biosynthesis and metabolism during the period at which the worm development attains maturity (Yu J., et al., 2019). These data would explain the high expression of sja-miR-124-3p in 30-day-old worms in the suitable host (mice) compared with earlier developmental stages by the demand for high metabolism, growth rate, and egg laying at this stage. These results suggest that the sja-miR-124-3p would play an important role in regulating metabolism, growth, development, and ovulation of *S. japonicum*. However, a more detailed understanding of this mechanism needs further experimental verification.

Previous studies showed that miR-124-3p played a negative regulatory role during the process of oxidative stress, prevented the generation and accumulation of reactive oxygen species (ROS), and attenuated oxidative stress injury (Geng et al., 2017; Liu et al., 2020). ROS are produced and accumulated in the ovaries of mice during ovulation (Shkolnik et al., 2011). The level of oxidative damage increased during reproduction in female mammals and birds. Therefore, reproductive efforts are positively correlated with oxidative damage. Oxidative stress in females during egg production could damage specific proteins in eggs (Peter et al., 2008; Blount et al., 2016). Female worms lay many eggs on the 30th day after the infection. Simultaneously, worms consume energy to synthesize egg-related proteins, increasing the endogenous oxidative stress. These findings suggest that the high expression of sja-miR-124-3p in 30-day-old worms in mice in our study may be related to the regulation of protein synthesis and oxidative stress related to egg laying.

The inflammation in mice livers and lungs was not as serious as in the *M. fortis* or rats. The liver of *M. fortis* contained many inflammatory cells around schistosomes 10 days after infection with *S. japonicum* (Jiang et al., 2010). Sorci and Faivre (2009) reported that the pathogen-induced inflammation is closely related to increased ROS production, provoking immunological oxidative stress in the host. During schistosome infection, a large number of ROS are released by the host immune cells during aerobic metabolism to damage the worms. Thus, a high level of antioxidant enzyme transcription was detected in adult worms (de Paula et al., 2014). Worms are susceptible to oxidation-mediated killing and, thus, secrete antioxidants, such as glutathione peroxidase (GPX), glutathione-S-transferase (GST), and superoxide dismutase (SOD) (Loverde, 1998). These antioxidants enable parasites to escape the oxidative defense of host immune cells, surviving inside the host (de Paula et al., 2016). Studies have shown that glutathione peroxidase mRNA is also one of the target genes of miR-124-3p (Valeria et al., 2020). Microarray analysis results showed that *S. japonicum* derived from rats demonstrated growth retardation and pathological change, which may be related to distinctive differential expression associated with development, growth, metabolism, redox, and signal transduction-related genes (Peng et al., 2011a; Peng et al., 2011b). Here, upregulating sja-miR-124-3p may inhibit the secretion of antioxidant enzymes in worms and affect the growth and development of worms.

To further clarify the regulatory role of sja-miR-124-3p in the growth and development of *S. japonicum*, we used mice as an animal model in this study. sja-miR-124-3p agomir was injected through the tail vein 9 times with 3-day intervals beginning from day 13 post-infection. Although the current study did not find statistically significant differences in worm recovery, male and female worms became shorter, the width of male worms decreased, and the outer circular muscle and the male posterior part had a damaged surface. The width of female worms increased, the development of vitelline cells was abnormal, and egg laying was reduced. The egg number, the granuloma area formed by a single schistosomal egg in the host liver, the pathological changes, and the hatching rate of liver eggs were reduced. Tail vein injection of sja-miR-124-3p agomir in mice resulted in altered worm structure and decreased oviposition rate, suggesting that a balanced expression of sja-miR-124-3p is required for normal worm development and reproduction. The reduced pathological effect might be related to the lower egg production and the hindrance of the host’s ROX by the overexpressed sja-miR-124-3p. Together, these results suggest that sja-miR-124-3p overexpression in mice affected not only worm growth and development but also egg laying and hatching.

Excessive ROS accumulation damaged oocytes and luteinized granulosa cells, resulting in lower fertilization, pregnancy rate, and embryo quality (Peng et al., 2011b; Karuputhula et al., 2013; Jia et al., 2019). Oxidative stress may cause abnormal meiosis, lower fertilization rate, delayed embryonic development, and reproductive disease. Follicle numbers and oocyte quality were affected (Jia et al., 2019). The p38 MAPK regulates homeostasis upon oxidative stress. RNA interference (RNAi) of Smp38 MAPK *in vitro* resulted in decreased egg production and ovary area, death of most eggs before reaching maturity, damaged adult worm tegument, and underdeveloped ovaries in females (de Paula et al., 2014; Avelar et al., 2019). In this study, after sja-miR-124-3p agomir was continuously injected into mice several times, the pathological changes ameliorated in the host liver. These results suggest that the high-level sja-miR-124-3p of worms from non-susceptible hosts may be related to exogenous oxidative stress caused by the strong immune response.

The miR-124-3p is widely distributed in many tissues but mainly expressed in neurons in the developing and adult nervous system. This microRNA is highly abundant in neuronal exosomes and plays an important role in nervous system development (Yu et al., 2008; Yelick et al., 2020). Schistosomes have a complex and well-developed nervous and sensory system that supports their response to many environmental stimuli throughout their life cycle (Liu et al., 2022). Studies have shown that anachronism, which encodes a secreted inhibitor of neuroblast proliferation, is regulated by miR-124. miR-124 maintained the proliferation of neural progenitor cells and regulated the generation of neurons during the development of *Drosophila* larvae (Weng and Cohen, 2012). The substances released by neurosecretory cells have been shown to regulate the development of oocytes in the ovary (Highnam, 1964). There are neuroendocrine cells in the ganglia of *S. japonicum*. Thus, we speculate that the sja-miR-124-3p regulates neurosecretory cells that would release substances to regulate the development of vitelline cells. This hypothesis might explain changes induced by injecting sja-miR-124-3p agomir in mice in our study, where the development of miracidia or egg maturation was hindered. There were decreases in liver egg number, in the hatching rate of liver eggs, and in the granuloma area formed by eggs.

The main pathological characteristics of hepatic fibrosis in schistosomiasis are the proliferation of hepatic stellate cells (HSCs) and the deposition of collagen type I and collagen type III (Chu et al., 2007). A study showed that activation of HSCs is critical during the early phase of hepatic fibrosis, and the antifibrotic drug rosiglitazone inhibits the activation of HSCs by upregulating miR-124-3p (Zhi et al., 2019). sja-miR-124-3p was detected in the serum and plasma of *S. japonicum*-infected hosts (Olioso et al., 2021; Zhu et al., 2021). miR-124-3p was conserved in different species, and the increasing level of sja-miR-124-3p in the host’s blood may inhibit the activation of host HSCs. Hence, we deduced that the regulation of sja-miR-124-3p on the host pathological damage should be not only *via* the effect on the development of reproductive organs and egg production of schistosome female worms but also through the regulation of the activation of HSCs and other immune relative cells of the host. This hypothesis might explain the milder liver pathological changes induced by schistosomes in non-susceptible hosts than in susceptible hosts. However, further experimental confirmation is still required.

After verification by dual-luciferase reporter assays, two potential target genes were identified, which were highly similar to ATP-dependent RNA helicase DDX1 (*sjDDX1*) and DNA polymerase II subunit 2 (*sjPOLE2*) after comparing the sequences through the NCBI and UniProt websites. We selected *sjDDX1* to carry out a further assessment in this paper. Previous studies showed that DDX1 mRNA was present at higher levels in fetal tissues of neural origin (retina and brain) than in other fetal tissues. DDX1 was distributed in RNA-transporting granules in dendrites of neurons as well as in astrocytes, cytoplasm, and nucleus (Godbout et al., 1998; Fang et al., 2005) and has also proven to be a key modulator in miRNA maturation and ovarian tumor suppression (Han et al., 2014). The distribution of miR-124-3p is highly similar to that of DDX1, suggesting that miR-124-3p may regulate the expression and biological function of DDX1. DDX1 null flies were infertile, significantly smaller, and developmentally delayed, with both oogenesis and spermatogenesis affected. Significantly smaller ovaries containing few to no mature eggs were observed in DDX1 null flies (Germain et al., 2015). DDX1 was highly expressed in the developing oocytes and the surrounding granulosa cells of the ovaries. Germline knockout of DDX1 resulted in embryonic lethality in mice (Hildebrandt et al., 2015; Hildebrandt et al., 2019). Similarly, our research showed that *sjDDX1* silencing affected worm survival and egg laying. Although there was no significant difference, *sjDDX1* silencing induced a 24.55% worm reduction rate and an 18.36% liver egg reduction rate. The area of egg granuloma in the livers was significantly decreased in the *sjDDX1* siRNA injection group.

## 5 Conclusions

In summary, this study revealed that the expression level of sja-miR-124-3p in *S. japonicum* from non-susceptible hosts was significantly higher than that of susceptible hosts. Mice consecutively injected with sja-miR-124-3p-agomir showed damaged worm structure and reduced host liver eggs, granuloma area, and host pathological changes. Interfering with the expression of *sjDDX1* (the target gene of sja-miR-124-3p) also partially affected the growth, development, and oviposition of *S. japonicum*. The current results emphasize the anti-developmental effect of sja-miR-124-3p agomir against schistosome. The balanced expression of sja-miR-124-3p is required for worm development and reproduction. We speculate that the sja-miR-124-3p regulates neurosecretory cells that would release substances to regulate the development of vitelline cells, and upregulating sja-miR-124-3p may inhibit the secretion of antioxidant enzymes in worms, thus affecting the growth and development of worms. Our results lay a foundation for further elaborating the regulatory role of sja-miR-124-3p in the development of *S. japonicum*.

## Data Availability Statement

The original contributions presented in the study are included in the article/**Supplementary Material**. Further inquiries can be directed to the corresponding authors.

## Ethics Statement

The animal study was reviewed and approved by the Animal Care and Use Committee of Shanghai Veterinary Research Institute, CAAS.

## Author Contributions

JL, YH, and GL conceived and designed the study. XZ, YH, and ZS performed the experiments. XZ and YH analyzed the data. XZ, JL, and GL wrote the paper. GL, YH, and AA revised the manuscript. All authors read and approved the final manuscript.

## Funding

This work was supported by the National Key Research and Development Program of China (http://www.most.gov.cn) (2017YFD0501306), National Natural Science Foundation of China (Grant No.31672541) and the innovation project of the Chinese Academy of Agricultural Sciences. The sponsor did not participate in the research design, data acquisition and interpretation, and manuscript preparation.

## Conflict of Interest

The authors declare that the research was conducted in the absence of any commercial or financial relationships that could be construed as a potential conflict of interest.

## Publisher’s Note

All claims expressed in this article are solely those of the authors and do not necessarily represent those of their affiliated organizations, or those of the publisher, the editors and the reviewers. Any product that may be evaluated in this article, or claim that may be made by its manufacturer, is not guaranteed or endorsed by the publisher.
