# Supplementary material for: The Potential Role of MicroRNA‐124‐3p in Growth, Development, and Reproduction of Schistosoma japonicum
Source: Front Cell Infect Microbiol. 2022 Apr 13;12:862496. doi: 10.3389/fcimb.2022.862496 (PMC9043613; doi:10.3389/fcimb.2022.862496)
Supplement: Supplementary file 1 [file Table_1.docx]

**Supplementary Table 1.** Animal infection and samples collection.

| The timing of worm collection | No. of cercariae infected per mice | No. of cercariae infected per rats |
| --- | --- | --- |
| 10 d P.I. | 200 | 2000 |
| 20 d P.I. | 100 | 1000 |
| 30 d P.I. | 60 | 600 |
| 40 d P.I. | 40 | 400 |

P.I.: post infection.
