# Supplementary material for: The Potential Role of MicroRNA‐124‐3p in Growth, Development, and Reproduction of Schistosoma japonicum
Source: Front Cell Infect Microbiol. 2022 Apr 13;12:862496. doi: 10.3389/fcimb.2022.862496 (PMC9043613; doi:10.3389/fcimb.2022.862496)
Supplement: Supplementary file 2 [file Table_2.docx]

**Supplementary Table 2.** The regulating role of sja-miR-124-3p on the development of *S. japonicum* in mice.

| Group | female burden (mean ± SD) | male burden (mean ± SD) | worm burden (mean ± SD) | developmental rate |
| --- | --- | --- | --- | --- |
| sja-miR-124-3p agomir | 12± 1.41 | 16.25± 4.11 | 26.75± 6.29 | 66.88% |
| sja-miR-124-3p antagomir | 10.75± 1.89 | 15.75± 6.29 | 26.5± 5.45 | 66.25% |
| NC | 11.6± 3.36 | 13.8± 4.35 | 25.4± 6.50 | 63.50% |
| PBS | 12.8± 1.64 | 13.8± 1.30 | 26.6± 2.70 | 66.50% |
