# Supplementary material for: The Potential Role of MicroRNA‐124‐3p in Growth, Development, and Reproduction of Schistosoma japonicum
Source: Front Cell Infect Microbiol. 2022 Apr 13;12:862496. doi: 10.3389/fcimb.2022.862496 (PMC9043613; doi:10.3389/fcimb.2022.862496)
Supplement: Supplementary file 3 [file Table_3.docx]

**Supplementary Table 3**. The regulating role of sja-miR-124-3p on liver egg production and egg hatching.

| Group | Liver weight (g) (mean ± SD) | Eggs count (mean ± SD) | EPG (mean ± SD) | Miracidium count (mean ± SD) | Hatchability (mean) | Reduction of hatching rate (mean) | ERR |
| --- | --- | --- | --- | --- | --- | --- | --- |
| sja-miR-124-3p agomir | 2.208 ± 0.248** | 116977. 8±38313.2 | 60837.62±17288.22* | 2088.9±6012.3* | 1.828%* | 75.84% | 36.97%* |
| sja-miR-124-3p antagomir | 1.725 ± 0.243 | 115377.8±33874.4 | 73511.09±10289.35 | 5022.2±1260.2 | 4.396% | 41.90% | 23.84% |
| NC | 1.734 ± 0.178 | 140089.0±46400.3 | 92392.06±29863.37 | 3022.2±154.0* | 2.330%* | 69.21% | 4.28% |
| PBS | 1.602 ± 0.242 | 107111.1±33762.9 | 96520.66±25660.59 | 11955.6±4598.2 | 7.566% | - | - |

EPG: The number of eggs per gram liver. ERR: The egg reduction rate. Hatchability: the liver egg hatching rate.

Statistically significant differences are shown by * (*p* < 0.05), ** *(p* < 0.01).
