# Supplementary material for: The Potential Role of MicroRNA‐124‐3p in Growth, Development, and Reproduction of Schistosoma japonicum
Source: Front Cell Infect Microbiol. 2022 Apr 13;12:862496. doi: 10.3389/fcimb.2022.862496 (PMC9043613; doi:10.3389/fcimb.2022.862496)
Supplement: Supplementary file 4 [file Table_4.docx]

**Supplementary** **Table 4.** The effect of *sjDDX1* on the development, egg production of *S japonicum* in mice by RNA interference.

| Group | worm burden (mean ± SD) | developmental rate | worm reduction rate | Liver weight (g) (mean ± SD) | EPG (mean ± SD) | ERR | Miracidium count (mean ± SD) | Hatchability (mean) | Reduction of hatching rate (mean) |
| --- | --- | --- | --- | --- | --- | --- | --- | --- | --- |
| *sjDDX1* siRNA | 42.00 ± 15.62 | 52.50% | 24.55% | 4.34±0.54 | 30504.62±7552.57 | 18.36% | 11022.2±2977. 2 | 8.60% | 0% |
| Irrelevant siRNA | 46.67 ± 10.26 | 58.33% | 16.17% | 4.73±0.52 | 33881.72±8454.17 | 9.32% | 7600±3711.2 | 5.15% | 30.10% |
| PBS | 55.67 ± 10.79 | 69.58% | - | 4.65±1.14 | 37364.9±13623.09 | - | 8555.6±6046.2 | 7.37% | - |

EPG: The number of eggs per gram liver. ERR: The egg reduction rate. Hatchability: the liver egg hatching rate.
